# Mud volcanoes and the presence of PAHs

**DOI:** 10.1038/s41598-020-58282-2

**Published:** 2020-01-27

**Authors:** Alexei Remizovschi, Rahela Carpa, Ferenc L. Forray, Cecilia Chiriac, Carmen-Andreea Roba, Simion Beldean-Galea, Adrian-Ștefan Andrei, Edina Szekeres, Andreea Baricz, Iulia Lupan, Knut Rudi, Cristian Coman

**Affiliations:** 1 Babeş-Bolyai University, Faculty of Biology and Geology, Department of Molecular Biology and Biotechnology, 1, M. Kogalniceanu Street, 400084 Cluj-Napoca, Cluj Romania; 2 Babeş-Bolyai University, Faculty of Biology and Geology, Department of Geology, 1, M. Kogalniceanu Street, 400084 Cluj-Napoca, Cluj Romania; 3 NIRDBS, Institute of Biological Research, 48 Republicii Street, 400015 Cluj-Napoca, Romania; 4 Babeş-Bolyai University, Faculty of Environmental Science and Engineering, 30 Fântânele Street, 400294 Cluj-Napoca, Romania; 5 Institute of Hydrobiology, Department of Aquatic Microbial Ecology, Biology Center of the Academy of Sciences of the Czech Republic, České Budějovice, Czech Republic; 6 Faculty of Chemistry, Biotechnology and Food Science, Norwegian University of Life Sciences, As, Norway

**Keywords:** Applied microbiology, Biodiversity

## Abstract

A mud volcano (MV) is a naturally hydrocarbon-spiked environment, as indicated by the presence of various quantities of PAHs and aromatic isotopic shifts in its sediments. Recurrent expulsion of various hydrocarbons consolidates the growth of hydrocarbonoclastic bacterial communities in the areas around MVs. In addition to the widely-known availability of biologically malleable alkanes, MVs can represent hotbeds of polyaromatic hydrocarbons (PAHs), as well - an aspect that has not been previously explored. This study measured the availability of highly recalcitrant PAHs and the isotopic signature of MV sediments both by GC-MS and δ^13^C analyses. Subsequently, this study highlighted both the occurrence and distribution of putative PAH-degrading bacterial OTUs using a metabarcoding technique. The putative hydrocarbonoclastic taxa incidence are the following: Enterobacteriaceae (31.5%), Methylobacteriaceae (19.9%), Bradyrhizobiaceae (16.9%), Oxalobacteraceae (10.2%), Comamonadaceae (7.6%) and Sphingomonadaceae (5.5%). Cumulatively, the results of this study indicate that MVs represent polyaromatic hydrocarbonoclastic hotbeds, as defined by both natural PAH input and high incidence of putative PAH-degrading bacterial OTUs.

## Introduction

Polyaromatic hydrocarbons (PAHs) represent a group of highly recalcitrant compounds. These compounds are formed due to either incomplete combustion of organic matter or geothermal catagenetic reactions^1,2^. PAHs are carcinogenic and 16 of them are considered priority pollutants by the United States Environmental Protection Agency^3,4^. Anthropogenic PAH pollution of soil is a widely spread concern across highly-industrialised areas. Implementation of effective countermeasures against this environmental issue may minimise the incidence of PAH-induced cancer among local populations^1,5^.

Soil is a natural sink for both petrogenic and pyrogenic PAHs. Their soil degradation depends on their intrinsic molecular weight. High-molecular-weight (HMW) PAHs (≥4 benzene rings) tend to be retained by both organic and clay-derived fine particles. Meanwhile, the sinking of low-molecular-weight (LMW) PAHs (≤3 benzene rings) is unlikely due to their ability to volatilize at room temperature^6–9^. Hence, green and inexpensive methods that can provide high degradation rates of soil-trapped HMW PAHs are of great interest.

Bacterial bioremediation of hydrocarbon-contaminated soils has been studied because of its cost-effectiveness^10^. Some research groups have elucidated the essential characteristics of bacterial PAH consumption in soil by describing Proteobacterial taxa as PAH-degrading markers^11,12^. However, these studies have a common feature: investigation of bacterial communities isolated from soil that has been recently contaminated with industrially derived PAHs^13,14^. No study to date has attempted to link PAH bacterial consumption with naturally hydrocarbon spiked environments, such as mud volcanoes (MVs).

The genesis of petrogenic PAHs depends both on subsurface movements of sediments, fluids, and kerogen maturation processes. During kerogen maturation, hydrogen distribution determines the accumulation of significant quantities of hydrogen-rich compounds, such as methane: the cornerstone element of MV genesis. In addition to volatile LMW alkanes, HMW PAHs are formed as by-products of kerogen maturation reactions^15,16^.

Most recent studies have focused on the influence of LMW alkanes on the distribution and metabolic behaviour of MV microbial communities. All of these studies used both culture-dependent (enrichment cultures) and culture-independent techniques (*mcr*A and 16S rRNA gene profiling, as well as metagenomics)^17–20^. However, little attention has been paid to the polyaromatic aspects of MV.

This study investigated sediments that were sampled from previously undescribed MVs in the Republic of Moldova. The study focused on PAH availability, isotopic signature, and mineralogical profile of the MV sediments (i.e., mud). Additionally, the study highlighted the occurrence and distribution of putative PAH-degrading bacterial operational taxonomic units (OTUs).

## Results

### Physicochemical profile

The investigated MV sediments were clays. These clays had the following physicochemical features: pH = 8.70; Eh = −115 mV; salinity = 0.3%; electrical conductivity = 713–1620 mS/cm. X-ray diffraction indicated that sediments are composed mainly of quartz (JCPDF 1-079-1910), calcite (JCPDF 00-002-0629), muscovite (JCPDF 00-002-0467), and feldspar (JCPDF 00-002-0457 and JCPDF 00-018-1202). The sediments sampled from the surface of MV were aerated. The sediments sampled from the 10–80 cm depth were saturated with water and depleted of oxygen.

The PAH concentration in the mud varied with depth; higher values were observed on the MV surface (33.44 µg/kg) followed by a 4- to 7-fold decrease at depths ≥10 (cm) (5.55 µg/kg) (Table 1).

**Table 1:** Concentration of polyaromatic hydrocarbons (PAHs) and TPH (Total Petroleum Hydrocarbons) concentration of the mud samples collected from both surface and 10–80 cm depth.

PAHs	PAHs concentration (µg/kg)				
	Surface	−10 cm	−20 cm	−40 cm	−80 cm
Naphthalene	0.87	0.48	0.30	0.29	0.93
Fluorene	0.82	0.63	0.39	0.36	0.40
Phenanthrene	2.70	1.24	0.28	0.52	0.85
Fluoranthene	1.41	1.52	0.26	0.24	0.38
Pyrene	2.28	2.09	0.18	1.27	1.46
Benzo[b]fluoranthene	12.26	12.02	2.18	2.28	1.89
Benzo[a]pyrene	6.62	4.63	—	—	1.86
Indeno(1,2,3-cd)pyrene	2.77	0.87	—	—	0.37
Dibenz[a,h]anthracene	1.54	1.43	0.38	0.59	—
Benzo[ghi]perylene	1.24	1.34	0.99	—	—
Total PAH	33.44	28.29	4.95	5.55	8.22
TPH (mg/kg)	1.37	1.43	1.20	0.80	0.90

We observed a difference between HMW and LMW PAH concentrations in the investigated mud samples. The HMW PAHs such as benzo[b]fluoranthene (12.26 µg/kg) and benzo[a]pyrene (6.62 µg/kg) showed higher concentrations on the surface of the MV. The concentrations of LMW such as phenanthrene and pyrene reached 2.70 and 2.28 µg/kg, respectively. Mud samples collected from the ≥10 cm depth had negligible LMW PAH concentrations compared to surface MV samples. Total petroleum hydrocarbon (TPH) values reached 1.43 mg/kg (Table 1).

**Figure 1:**
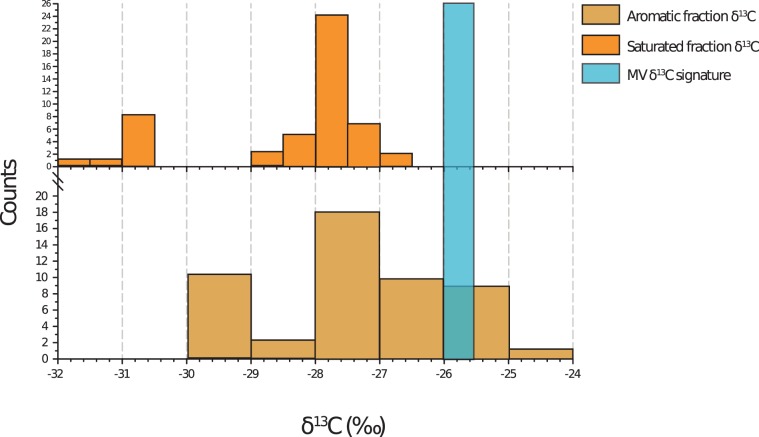
Histograms of (MV) mud volcano stable carbon isotope values. Values of saturated and aromatic fractions extracted from various crude oil spiked sediments^24–27^.

The analysis of δ^13^C indicated a narrow interval of values attributed to the MV mud samples. The values varied between −25.52 and −26.00‰ (average = −25.71‰, SD = 0.18‰). The δ^13^C analysis underlined the isotopic predominance of the aromatic fraction over the aliphatic fraction (Fig. 1).

### Microbial community profiles

To provide an accurate OTU profile, we described the features of each mud sample. Notably, due to the low resolution of the metabarcoding procedure, we relied on risk pooling strategy^21^, “*reducing overall risk of erroneous statement by combining incidence of variables of interest*” when reporting any *“putative PAH-degrading bacterial taxa”* statements extrapolated from metabarcoding data^21^.

**Figure 2:**
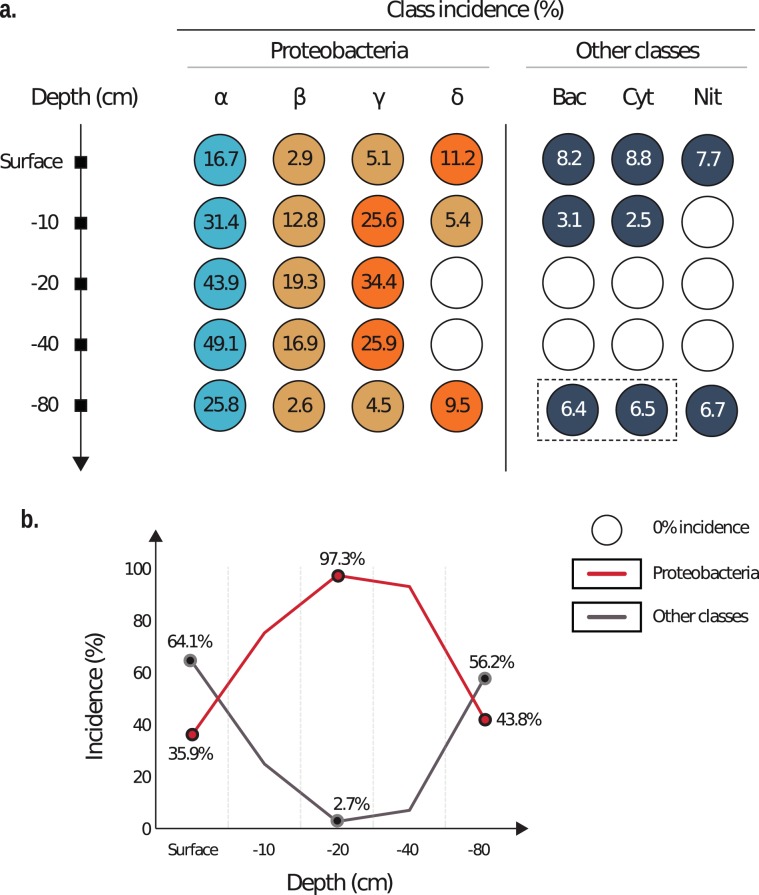
Contrast between the incidence of the mud volcano operational taxonomic units linked to Proteobacteria classes and non-Proteobacteria units within the investigated mud samples. (**a**) Explicit bacterial class incidence. (**b**) Overall Proteobacteria incidence trendline. Note: Nit – Nitriliruptoria; Instead of Bac - Bacilli and Cyt - Cytophagia incidence, dashed rectangular includes incidence of Anaerolineae and Acidimicrobiia classes, respectively.

The MV surface sample had the most diverse OTU profile. This sample had the highest richness and phylogenetic diversity among all the collected samples (Table 2). The abundance of non-Proteobacterial units exceeded that of Proteobacteria. Bacterial classes with the highest abundance were Bacilli, Cytophagia, and Nitriliruptoria (Fig. 2a). Meanwhile, the orders with the highest OTU incidence were *Actinomycetales* (5.7%), *Myxococcales* (5.2%), *Rhodobacterales* (4.5%), *Acidimicrobiales* (4.1%), *Rhodospiriralles* (3.0%), and *Xanthomonadales* (1.3%).

**Table 2:** Bacterial α-diversity of the investigated mud samples.

Depth (cm)	Shannon Index	PD^*^ whole tree	Chao1	Observed Species	Simpson Index
Surface	7.7	32.5	506.7	472.0	1.0
−10	6.0	29.2	423.0	399.0	0.9
−20	3.2	10.5	97.3	84.0	0.8
−40	4.0	13.9	162.4	137.0	0.9
−80	7.7	31.7	506.8	446.0	1.0

^*^PD - phylogenetic diversity.

The samples collected from the 10 cm depth were characterised by lower richness, contrary to the surface. However, this depth was defined by high bacterial diversity (Table 2). As shown in Fig. 2a the most abundant orders were *Rhizobiales* (24.6%), *Enterobacteriales* (20.4%), *Burkholderiales* (11.1%), *Sphingomonadales* (3.2%), and *Xanthomonadales* (1.8%).

The samples collected from the 20 cm depth had the lowest richness and diversity (Table 2). Alphaproteobacteria and Gammaproteobacteria representatives dominated the overall profile. Contrary to the surface and 10 cm samples, the abundance of Deltaproteobacteria OTUs decreased to a minimum of 0.3%. Classes that predominated on the surface and 10 cm depth samples seemed not to have any substantial impact on the local community, except for Proteobacteria units (Fig. 2a). The 20 cm sample was dominated by such orders as *Rhizobiales* (38.8%), *Enterobacteriales* (31.5%), *Burkholderiales* (19.2%), and *Sphingomonadales* (3.6%).

The OTU profile attributed to the 40 cm sample had similar features to the 20 cm sample (Table 2). In this study, Alphaproteobacteria, Gammaproteobacteria, and Betaproteobacteria units had the highest abundances from all the samples investigated in this study: 49.9%. 25.9% and 16.9%, respectively (Fig. 2a). The most abundant orders were *Rhizobiales* (39.1%), *Enterobacteriales* (22.1%), *Burkholderiales* (16.9%), *Sphingomonadales* (8.0%), and *Rickettsiales* (1.6%).

Mud sampled from the depth of 80 cm had similar richness and diversity which was previously observed on the surface (Table 2). In this study, we observed that the OTU profile was represented by non-Proteobacteria units (56.2%). The incidence of Proteobacteria marked a twofold decrease relative to the values observed in the 40 cm samples. Nevertheless, this value was higher than the values observed on the surface level (Fig. 2b).

**Figure 3:**
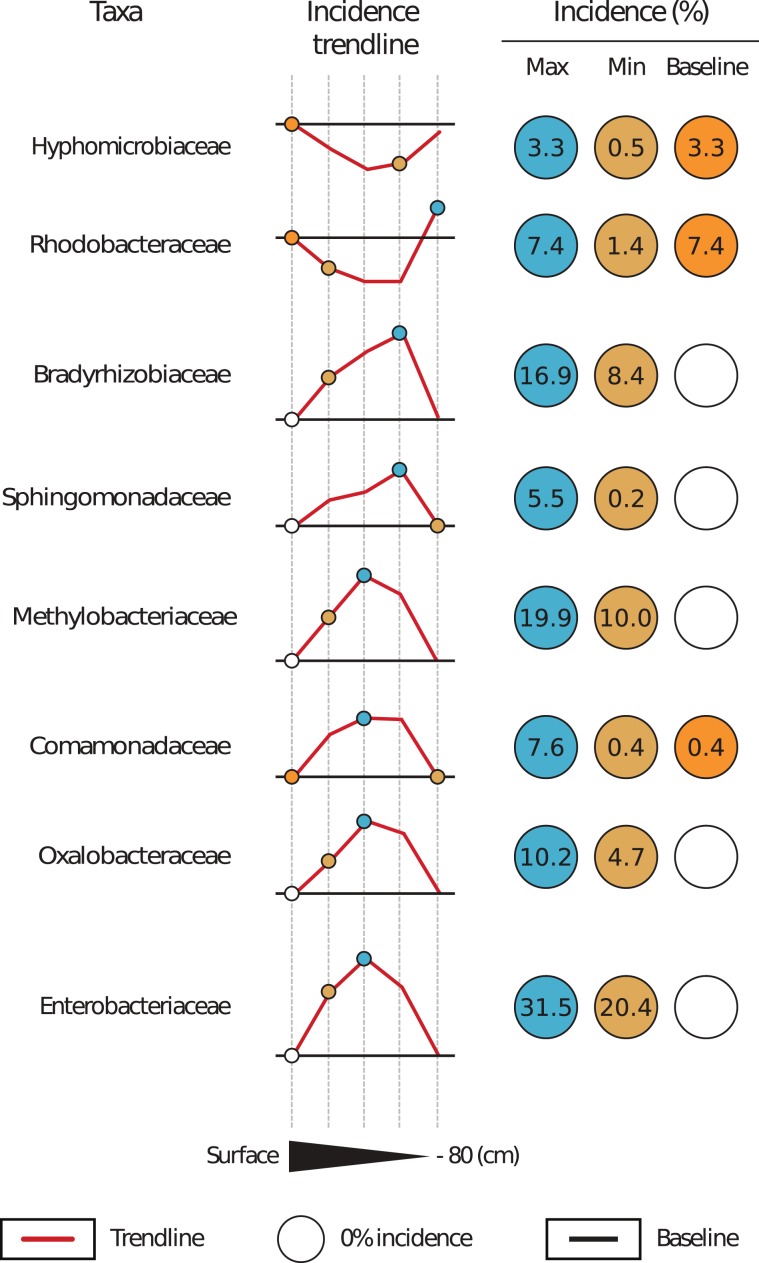
Incidence of mud volcano operational taxonomic units linked to PAH-degrading bacteria within investigated mud samples. Note: Baseline value is equal to the surface taxa incidence; Max – maximum taxa incidence; Min – minimum taxa incidence ≠ 0%.

The metabarcoding of the overall MV bacterial profile highlighted the predominance of Proteobacteria OTUs in all the investigated mud samples except for the surface sample (Fig. 2b). OTUs linked to PAH-degrading bacteria followed this trend. The majority of the OTUs had incidence values as high as 77.8–87.4%. The lowest incidence of PAH-degrading representatives could be observed on the surface, where only Hyphomicrobiaceae and Rhodobacteraceae played a significant role in the local community. Generally, the overall OTU library was dominated by Enterobacteriaceae (31.5%), Methylobacteriaceae (19.9%), Bradyrhizobiaceae (16.9%), Oxalobacteraceae (10.2%), Comamonadaceae (7.6%), and Sphingomonadaceae (5.5%) (Fig. 3).

## Discussion

GC-MS analysis showed that MV was spiked with PAHs. The dominance of HMW PAHs, such as benzo[b]fluoranthene and benzo[a]pyrene on the MV surface was terminated by low solubility, constant asphaltene expelling, and limited bacterial ability to oxidise polyaromatic compounds^15,22^. However, scarce LMW PAHs on the MV surface were caused by rain washing, summer-time volatilisation or extensive bacterial oxidation. The PAH traces that reached the lower hypoxic mud strata were slowly mineralized by bacteria or fungi^23^. Cumulatively, these processes could explain the decreasing gradient of PAHs.

To highlight the aliphatic/aromatic isotopic shift in the investigated samples, the MV δ^13^C signature was compared to the other hydrocarbon-spiked sediment signatures^24–27^. The results of this procedure underlined an overall δ^13^C shift towards aromatic petroleum fractions. This aromatic shift was caused by both underlying catagenesis and the predominance of aromatic compounds in the MV^15,16^. The aromatic shift was consistent with the recurrent encounter of PAHs and TPHs in the MV samples.

Proteobacteria OTUs outclassed non-Proteobacteria OTUs at depths of 10–40 cm. The majority of the encountered Proteobacteria units were linked to the well-known PAH-degrading bacterial taxa. The high incidence of the putative Proteobacteria PAH-degrading taxa could be attributed to the MV hydrocarbon-spiked medium.

The inner 10–40 cm MV samples were dominated by *Methylobacterium* sp., a well-known phenanthrene-degrading genus^28^. *Ralstonia* sp. was frequently observed in the investigated samples. *Ralstonia* can oxidise HMW PAHs such as pyrene^29^. Sphingomonades were identified in the 10–40 cm-depth samples. Two genera mainly represented the group: *Novosphingobium* sp. and *Sphingomonas* sp., which are known to degrade various PAHs due to their substrate unspecific dioxygenases^30,31^. Bradyrhizobiaceae had a significant presence in the 10–40 cm interval. Some of the representatives of this family can degrade pyrene^32^. However, high Bradyrhizobiaceae incidence could be caused by bacterial/plant symbiotic nitrogen-fixation processes^33^. Comamonadaceae were also observed in the mud samples. Comamonadaceae genera, such as *Delftia* and *Acidovorax* are well-known PAH-degraders^34,35^. A high incidence attributed to Enterobacteriaceae was observed. Enterobacteriaceae are considered to be bioindicators for highly PAH amended soils and they are known to degrade PAHs with up to 4 aromatic rings^12,13^.

The surface and 80 cm samples were not dominated by putative hydrocarbonoclastic OTUs. PAH-degrading taxa were Hyphomicrobiaceae and Rhodobacteraceae. The former group includes bacteria with an enzymatic package aimed at downstream degradation of fluoranthene^36^. The latter does not have any hydrocarbonoclastic abilities but can still dominate bacterial communities in ecosystems contaminated with crude oil^37^.

This study investigated the PAH availability and isotopic signature of MV sediments. Both GC-MS and δ^13^C results confirmed that MV sediments are spiked with PAHs. Furthermore, metabarcoding of MV indigenous bacteria provided a library with an overwhelmingly high abundance of OTUs linked to well-known PAH-degrading bacterial taxa, such as Enterobacteriaceae (31.5%), Methylobacteriaceae (19.9%), Bradyrhizobiaceae (16.9%), Oxalobacteraceae (10.2%), Comamonadaceae (7.6%) and Sphingomonadaceae (5.5%). Cumulatively, these results indicate that MVs can be veritable polyaromatic hydrocarbonoclastic hotbeds. However, subsequent studies should be performed. These studies must address the influence of HMW alkanes and obtain a more accurate snapshot of the local bacterial community. Nevertheless, the obtained data suggest that MVs could represent a polyararomatic environment, which may serve as a platform for further PAH bioremediation studies.

## Methods

### Experimental design

**Figure 4:**
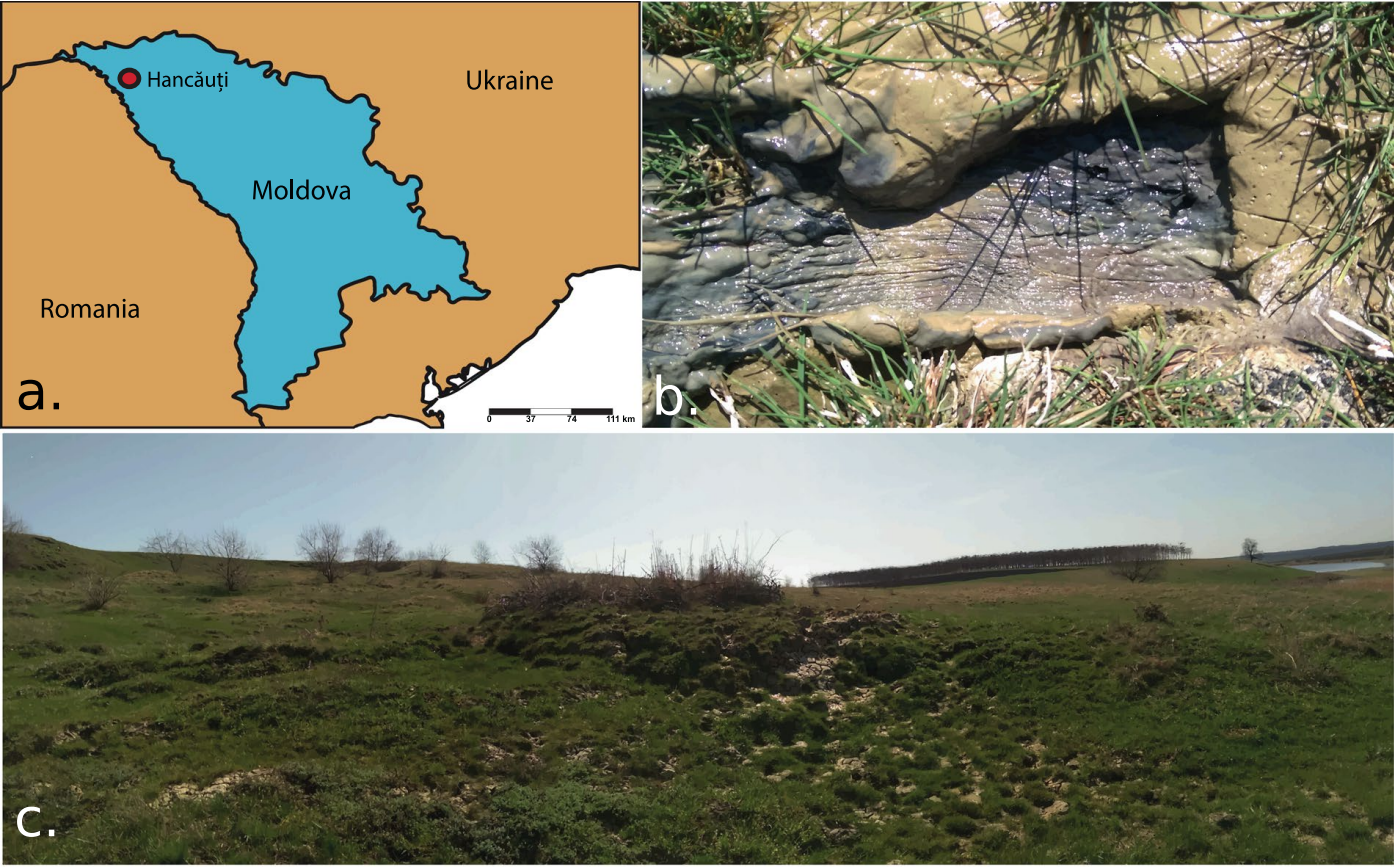
Geographical localisation of the investigated mud volcano (MV). (**a**) MV (Hancăuți) geographical localization. (**b**) Visual aspect of the investigated mud samples, (**c**) MV panoramic view.

Investigated MV is situated in Hancăuți, the Republic of Moldova (Lat: 48.036837°N, Long: 27.196834°E) (Fig. 4). MV clay-derived sediments with water content were collected in two distinct batches. Mud samples were collected in triplicate using a push-tube sampler (300 g per sample). The mud samples were carefully taken from both MV surface crust and four successive depths (qualified as inner samples) within the MV cone: 10, 20, 40, and 80 cm. After the sampling procedure, mud samples were rapidly transported on ice to the laboratory^38^. The first batch of mud samples, kept at −20 °C, was subjected to microbial diversity analysis which implied metabarcoding of the overall MV bacterial community. The second batch of samples, kept at 4 °C, was chosen for pH, salinity, conductivity, GC-MS and mineralogical analyses. Before all analyses, equal quantities of mud from each set of triplicates were mixed to form a representative sample. Notably, data obtained from both of the investigated batches cannot be correlated because MV is not uniformly stratified. This feature comes from intermittent MV activity and underlying downhill relief.

### Physicochemical and mineralogical profiles

Physicochemical parameters (pH, salinity, redox potential, electrical conductivity) were measured on site using a portable multi-parameter Multi 3320 WTW. X-ray diffraction analysis of powder samples was carried out on a Bruker D8 Advanced in Bragg-Brentano configuration using a Cu anode X-ray source operated at 40 kV and 40 mA. In the beam path was a 0.6 mm slit, Ni 0.0125 mm filter, and on receiving side a 2.5° Soller slit and a LynxEye detector. The X-ray diffraction was recorded from 5° to 64° (2 T) with 0.02° (2θ) step and 0.5 s/step. DifracEva software (Bruker Co.) and the ICDD database are used for mineral identification.

### DNA extraction and microbial biodiversity investigation

DNA metabarcoding analysis was performed to describe an accurate bacterial profile of the investigated MV. Metagenomic DNA was extracted from the mud samples using the ZR Soil Microbe DNA MiniPrep^TM^ kit (ZymoResearch, USA) according to the manufacturer’s instructions. The replicate DNA samples from each depth were quality checked on a 1% agarose gel (data not shown) and pooled followed by quantification using a NanoDrop spectrophotometer (Thermo Scientific, Wilmington, DE, USA) (data not shown). These DNA samples were stored at −20 °C until further analysis. Microbial diversity investigation was performed based on a metabarcoding approach using the V3-V4 regions of the 16 S rRNA gene. DNA fragments were amplified through PCR with the PRK341F/PRK806R universal primer pair^39^. The PCR reaction mixture (final volume 25 μl) for each reaction included 1X HOT FIREPol® PCR mix (Solis BioDyne, Estonia), 200 nM uniquely tagged forward and reverse primers, 1 μl of sample DNA and 18 μl water. The reaction conditions included an initial denaturation at 95 °C for 15 min followed by 25 cycles of 95 °C for 30 s, 50 °C for 30 s, 72 °C for 45 s, ending with a final elongation at 72 °C for 7 min. The Qubit® dsDNA HS Assay Kit and the Qubit® Fluorometer (Life Technologies, USA) were used to measure the concentration of amplicons. Equal amounts of amplicons were pooled into a normalised library. The PerfeCta® NGS Quantification Kit from Illumina (Quanta BioSciences, USA) was used to determine the library concentration. Afterwards, the sequencing library was diluted in Tris pH 8.5 to a final concentration of 4 nM. Sequencing was performed on a MiSeq platform (Illumina, USA) using V3 sequencing chemistry with 300-bp paired-end reads.

The raw sequencing data (SRA: SRP133053) were processed as described by Chiriac *et al*.^40^. Briefly, singletons with less than 350 nucleotides and those with more than 0.2 total expected errors were discarded using the Usearch v8 pipeline^41^. *De novo* and reference chimaera checking were carried out with Usearch v8, using the latest version of the Greengenes database (‘13_8’) as a ref. ^42^. Taxonomic affiliation of representative OTUs was established in QIIME^43^, and the plastidial and mitochondrial sequences were filtered out from the final OTUs table.

### GC-MS assay

GS-MS analysis was performed to evaluate hydrocarbon availability in the investigated samples. A combination of existing methods^38,44–46^ was used for mud sampling and processing. A total of 3 (g) of dried, ground and sieved (500 µm) mud sample was extracted by sonication (3 × 15 min) with CH_2_Cl_2_: n-C_6_H_6_ (1:1 v/v) (3 × 10 ml). The collected extracts were passed through Na_2_SO_4_ anhydrous cartridges, previously washed with n-C_6_H_6_ and CH_2_Cl_2_, then evaporated to dryness and redissolved in 2 ml CH_2_Cl_2_: n-C_6_H_6_ (1:1 v/v) for 16 priority PAH analyses and in 1 ml CH_2_Cl_2_: n-C_6_H_6_ (1:1 v/v), for total petroleum hydrocarbon (TPH) analysis^47^.

PAH and TPH analyses were performed by GC-MS using a Focus GC gas chromatograph with a DSQ II quadrupole mass spectrometer detector (Thermo Electron Corporation, USA). GC separation was achieved on a TR-5MS non-polar capillary column (30 m × 0.25 mm × 0.25 µm) (Thermo Electron Corporation, USA) containing 5% phenyl polysilphenylene-siloxane. Samples were injected (1 µl) in splitless mode by an autosampler Thermo TriPlus AS. Transfer line and ion source temperatures were 300 °C and 200 °C for PAH analysis and 310 °C and 200 °C for TPH analysis. The oven temperature programme started at 120 °C up to 300 °C for PAH analysis and from 100 °C up to 310 °C for TPH analysis. Helium is used as the carrier gas at a flow rate of 1.2 ml/min. Selected ion monitoring mode was used during GC-MS analysis for PAH and full scan mode for TPH analysis^38,44–47^.

### δ^13^C analysis

Analysis of δ^13^C was performed to identify the MV isotope signature. Mud samples were dried at 40 °C, milled, and homogenised in an agate mortar. To remove any carbonates from the samples^48^, milled material was treated with 10% HCl^49^ until no reaction was observed and then left for 24 hours in the acid solution. The resulting material was dried at 40 °C for 48 hours and then milled and homogenised again in an agate mortar. The agate mortar was washed and treated with hydrogen peroxide between each sample and then dried.

Isotopic δ^13^ values were measured using a Picarro G2121-i type analyser (Picarro Inc., USA) coupled with a combustion module (Costech Analytical Technologies Inc., USA) in the Stable Isotope Laboratory of the Babeş-Bolyai University in Cluj-Napoca, Romania. Sample analysis was performed as previously described^50^. Measured values (two to four measurements on each sample) were calibrated using internal laboratory standards (atropine and acetanilide) and the average was reported. The δ^13^C values are expressed regarding VPDB^51^ using the following equation δ^13^C = [(R_sample_/R_standard_) − 1] × 1000 (‰), where R is the ^13^C/^12^C ratio. Precision was within ±0.09‰ (1σ) based on measured samples and the reproducibility of measurements was checked with measurements on standard B2155 (Elemental Microanalysis Ltd, UK), was within ±0.05‰ (1σ).

## Data Availability

Raw sequencing data generated during the current study are available in the SRA repository (SRP133053).
