# Author Correction: Mud volcanoes and the presence of PAHs

**DOI:** 10.1038/s41598-020-62513-x

**Published:** 2020-04-15

**Authors:** Alexei Remizovschi, Rahela Carpa, Ferenc L. Forray, Cecilia Chiriac, Carmen-Andreea Roba, Simion Beldean-Galea, Adrian-Ștefan Andrei, Edina Szekeres, Andreea Baricz, Iulia Lupan, Knut Rudi, Cristian Coman

**Affiliations:** 1 Babeş-Bolyai University, Faculty of Biology and Geology, Department of Molecular Biology and Biotechnology, 1, M. Kogalniceanu Street, 400084 Cluj-Napoca, Cluj Romania; 2 Babeş-Bolyai University, Faculty of Biology and Geology, Department of Geology, 1, M. Kogalniceanu Street, 400084 Cluj-Napoca, Cluj Romania; 3 NIRDBS, Institute of Biological Research, 48 Republicii Street, 400015 Cluj-Napoca, Romania; 4 Babeş-Bolyai University, Faculty of Environmental Science and Engineering, 30 Fântânele Street, 400294 Cluj-Napoca, Romania; 5 Institute of Hydrobiology, Department of Aquatic Microbial Ecology, Biology Center of the Academy of Sciences of the Czech Republic, České Budějovice, Czech Republic; 6 Faculty of Chemistry, Biotechnology and Food Science, Norwegian University of Life Sciences, Ås, Norway

Correction to: *Scientific Reports* 10.1038/s41598-020-58282-2, published online 27 January 2020

The original version of this Article contained errors.

The spelling of the author ‘Knut Rudi’ was incorrectly given as ‘Rudi Knut’.

As a result, in the Author Contributions,

“C.-A.R., S.B.-G., A.-Ș.A., E.S., A.B., I.L., R.K. and C.C. carried out the experimental work.”

now reads:

“C.-A.R., S.B.-G., A.-Ș.A., E.S., A.B., I.L., K.R. and C.C. carried out the experimental work.”

In addition, there was an error in Affiliation 6, which was incorrectly given as ‘Norwegian University of Life Sciences, 3 Universitet Student, 1430, Aas, Norway.’ The correct affiliation is listed below:

‘Faculty of Chemistry, Biotechnology and Food Science, Norwegian University of Life Sciences, Ås, Norway.’

These errors have now been corrected in the HTML and PDF versions of the Article.

